# Influences of an Aluminum Covering Layer on the Performance of Cross-Like Hall Devices

**DOI:** 10.3390/s16010106

**Published:** 2016-01-15

**Authors:** Fei Lyu, Xinfu Liu, Yinjie Ding, Eng-Huat Toh, Zhenyan Zhang, Yifan Pan, Zhen Wang, Chengjie Li, Li Li, Jin Sha, Hongbing Pan

**Affiliations:** 1 School of Electronic Science & Engineering, Nanjing University, Nanjing 210093, China; lvfeieric@gmail.com (F.L.); zhangzhenyan518@163.com (Z.Z.); wzwcwz@foxmail.com (Z.W.); lichengjie.520@163.com (C.L.); lili@nju.edu.cn (L.L.); shajin@nju.edu.cn (J.S.); 2 Globalfoundries Singapore, Singapore 738406, Singapore; XinFu.LIU@globalfoundries.com (X.L.); YINJIE.DING@globalfoundries.com (Y.D.); EngHuat.TOH@globalfoundries.com (E.-H.T.); 3 Nanjing Foreign Language School, Nanjing 210008, China; panyifanhappy@163.com

**Keywords:** cross-like Hall sensor, sensitivity, offset voltage, aluminum covering

## Abstract

This work studies the effects of an aluminum covering on the performance of cross-like Hall devices. Four different Hall sensor structures of various sizes were designed and fabricated. The sensitivity and offset of the Hall sensors, two key points impacting their performance, were characterized using a self-built measurement system. The work analyzes the influences of the aluminum covering on those two aspects of the performance. The aluminum layer covering mainly leads to an eddy-current effect in an unstable magnetic field and an additional depletion region above the active region. Those two points have influences on the sensitivity and the offset voltage, respectively. The analysis guides the designer whether to choose covering with an aluminum layer the active region of the Hall sensor as a method to reduce the flicker noise and to improve the stability of the Hall sensor. Because Hall devices, as a reference element, always suffer from a large dispersion, improving their stability is a crucial issue.

## 1. Introduction

In recent years, Hall effect sensors inducting magnetic fields have become increasingly indispensable in various fields, such as the manufacturing industry, consumer electronics, aerospace industry, and so on [1,2,3]. As a typical Hall device implementation, cross-shaped Hall sensors are considered more reliable, agile, miniaturizable and economical.

The sensitivity and offset are two important performance indicators of a Hall device. Hall sensors based on silicon processes always offer low sensitivities and suffer from large offsets. Among the impacting factors, the geometry has a significant effect on the sensitivity and offset [4,5,6,7,8,9]. In the past decade, Hall devices with various symmetrical geometries such as circle, square, octagon, cross-like, *etc.* have been designed and analyzed [10,11]. Hall sensors in a cross-like structure, with their high sensitivity and low offset, are considered to have the most bright prospects. Recently, various new Hall sensors structures were presented [12,13,14,15,16,17]. Most of the new ones are variants of the cross-like Hall sensors. For a cross-like Hall sensor, the most frequently used method in the design of the sensor to reduce the flicker noise [18] and to improve the stability of the Hall sensor [19] is by covering a P-type region or a metal layer on the active region of the Hall sensor. Our previous work has analyzed the sensitivity and offset of Hall sensors using the P-type region [20]. The intention of the present paper is to study the effects of the covering metal layer on the sensitivity and offset of the cross-like Hall sensor. There is a silicon dioxide layer between the covering metal and the active region and the covering metal must be connected to the ground while the Hall sensor is in operation. Due to the voltage between the metal and the active region of Hall sensor, a depletion region is formed under the silicon dioxide layer. This depletion region can effectively reduce flicker noise and improve the stability of the Hall sensor. Moreover, the metal layer has an influence on the sensitivity and offset. In this work, we use aluminum as the metal layer and the sensitivity and the offset of the cross-like sensor caused by the aluminum covering are studied in detail. We designed twenty Hall cells in four Hall chips with different structures. Section 2 introduces those designs, the test equipment and the measurement data. Section 3 analyzes the measurement data from two aspects: The sensitivity and offset. Section 4 summarizes all the work and gives the conclusions.

## 2. Design and Experiment

**Figure 1:**
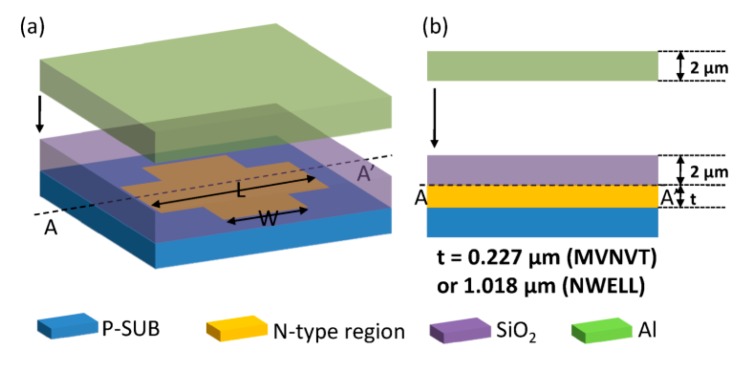
Schematics of the three-dimensional view (**a**) and sectional view (**b**) of the designed structures of Hall devices. L and W are the length and width of Hall device. S2 and S4 are covered by an aluminum layer, but S1 and S3 are not. The N-type region of both S1 and S2 is NWELL and S3 and S4 use MVNVT as the N-type region.

**Figure 2:**
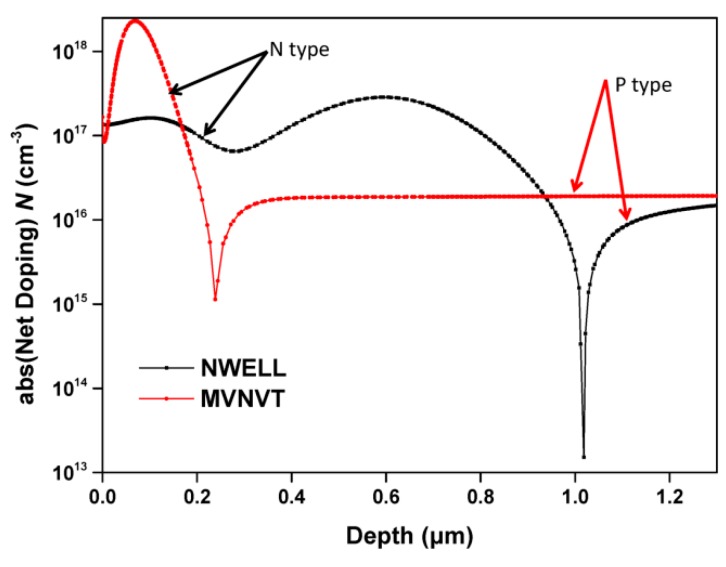
The doping profiles of NWELL and MVNVT.

Hall devices of four different structures have been designed and integrated in a 0.18 μm BCD lite^TM^ technology provided by Globalfoundries. Existing implants are employed to form the Hall devices without additional processes. Five Hall cells with the same structure and in different sizes are implemented in one Hall chip. Figure 1 illustrates the structures of the Hall devices we designed. These four structures all consist of an N-type region in a P-substrate, four contacts and a silicon oxide layer. The N-type region is used as the active zone of the Hall sensor. The four contacts are formed by N+ implantation symmetrically distributed at four branches of the Hall device. The current biases Hall sensor by one pair of opposite contacts and the other pair serve as the measurement contacts. The N-type regions of the first structure (S1) and the second one (S2) are NWELL, which is formed in the drain extension step of a medium voltage n-channel device. The third (S3) and the fourth structure (S4) replace NWELL by MVNVT, the process of low voltage p-channel device substrate. MVNVT is an N-type region with different doping profile of the NWELL and its doping profile is shown in Figure 2. Globalfoundries provided the doping profiles, which are obtained from the simulations using SILVACO Technology Computer Aided Design (TCAD). In addition, there is no special process above the silica layer in S1 and S3. However, S2 and S4are covered with an aluminum layer placed above the silica layer. The silica layer is used to isolate the metal layer and N-type region in order to eliminate the leakage current. The aluminum layer connected to the ground can generate a depletion region.

**Figure 3:**
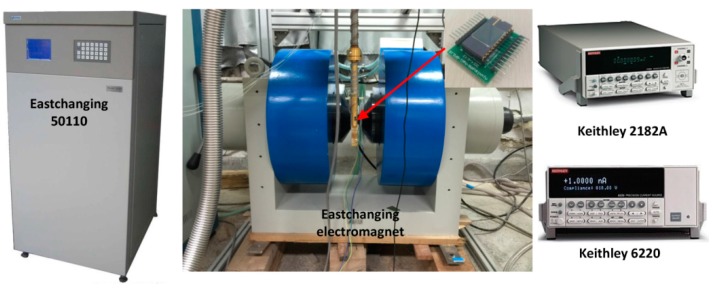
Self-built measurement system.

The measurement equipment used has been introduced in another recent paper of the authors [20] and is shown in Figure 3. An Eastchanging 50110 instrument (East Changing Technologies, Inc., Beijing, China) is responsible for applying a current to the electromagnet and a magnetic field we need appears at the center of electromagnet. The Hall device is put at the center of electromagnet perpendicular to the magnetic field. A Keithley 6220 device (Keithley Instruments, Inc., Cleveland, OH, USA) is used to bias the Hall device and a Keithley 2182A (Keithley Instruments, Inc., Cleveland, OH, USA) feeds back the voltage between two measurement contacts. At the room temperature, the biasing current is 0.1 mA and it remains unchanged. The value of magnetic field sampling 50 points varies from −1T to 1T.

## 3. Results and Discussion

### 3.1. Hall Device

**Figure 4:**
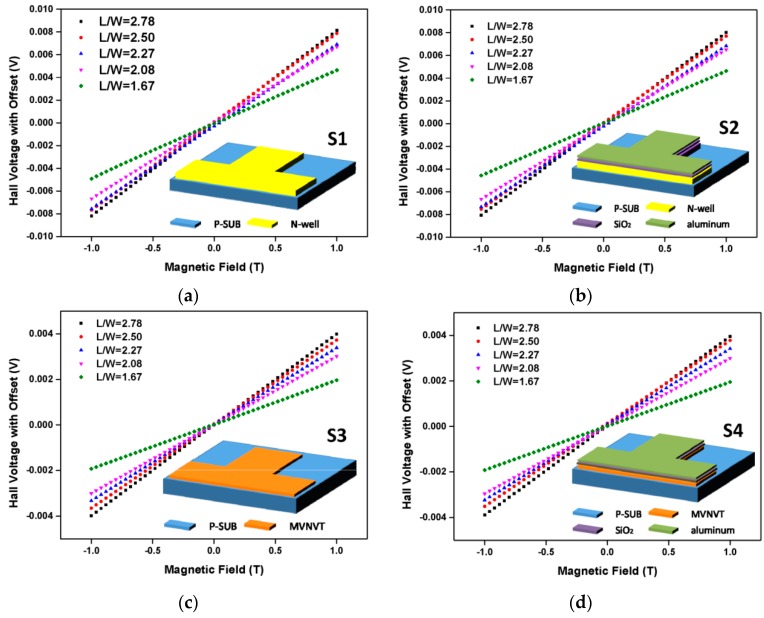
The measured Hall voltage (coupled with offset) as a function of applied magnetic field for S1 (**a**), S2 (**b**), S3 (**c**), S4 (**d**) with different L/W ratios.

When a current *I* is supplied through two biasing contacts and the Hall device is put in an orthogonal magnetic field *B*, the voltage *V* appears between the other contacts. This voltage contains two parts: Hall voltage *V_H_* and offset voltage *V_off_*. It can be expressed by:

(1) $$V=S_{I}IB+V_{off}$$

where *S_I_* is the current-related sensitivity. The measured voltage is this kind voltage expressed in above equation and illustrated in Figure 4. The current-related sensitivity *S_I_* of Hall device has the following analytical expression [21]:

(2) $$S_{I}=\frac{Gr_{H}}{nqt_{eff}}$$

Here, *G* is the correction factor depending on the geometry, *r_H_* is the Hall scattering factor, *t_eff_* is the effective depth of the N-type region, *n* is the doping concentration of the active region of Hall device and *q* is the elementary charge of an electron. L/W ratio that designers have most control of acts as a common influence factor of geometry and has been studied in a few papers [10,11].

**Figure 5:**
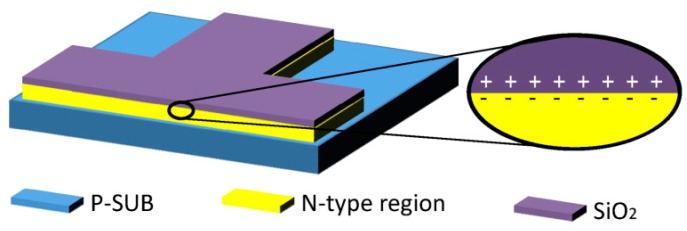
The surface effect on a CMOS Hall device.

As a reference component, it is taken for granted that the Hall device has excellent stability and low dispersion. However, because the uncertainty of some factors, CMOS Hall devices always suffer from dispersion caused by various reasons, such as the piezo-Hall effect and surface effects. There is always an oxide layer covering the Hall device. The oxide layer contains various amounts of charge carriers, which induce charges of opposite polarity in the Hall device's active zone as shown in Figure 5. The influence of the charge carriers in the oxide layer on the Hall sensors’ characteristics is called the surface effect. The surface effect impact on the current-related sensitivity and offset by the following equations [2]:

(3) $$\frac{\delta S_{I}}{S_{I}}=S_{I}\delta Q_{S}$$

(4) $$\delta B_{off}=\frac{S_{I}}{\mu_{H}}\delta Q_{S}$$

Here *δS_I_* is an instable part of the current-related sensitivity depending on the surface effect, *δQ_S_* is the variation in the interface charge density, *δB_off_* is an additional part of the offset depending on the surface effect, and *μ_H_* is the Hall mobility. In order to improve the stability of a CMOS Hall device, a method to eliminate the dispersion caused by surface effect is to get the Hall device’s active region away from the surface by introducing a depletion region. Improving the stability by covering a metal layer or a P-type region above the Hall device is very effective for this purpose.

### 3.2. Current-Related Sensitivity

**Figure 6:**
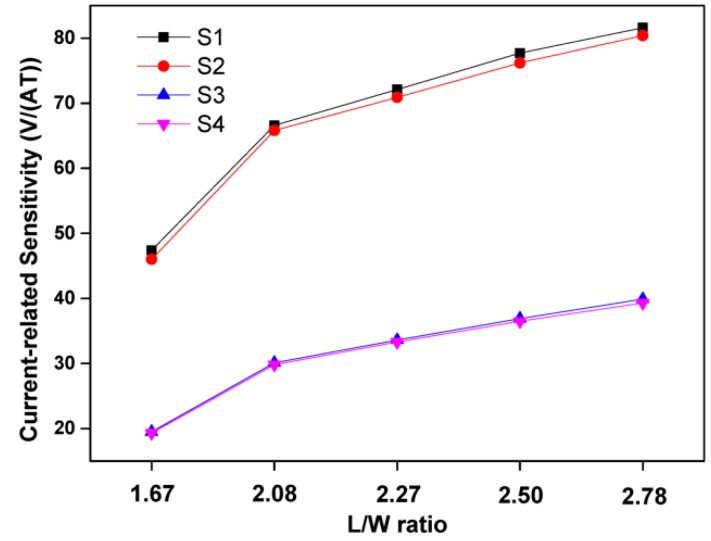
Relationship between L/W ratio and current-related sensitivity of S1, S2, S3 and S4.

According to Figure 6, S2 and S4 with an aluminum covering layer have worse current-related sensitivities compared to S1 and S3. Thanks to the paramagnetism of aluminum and the depletion region between the silicon dioxide layer and the active area of the Hall sensor, the sensitivity of the Hall sensor with an aluminum layer increases slightly. However the sensitivity moves in the opposite direction because of the eddy current effect in an unstable magnetic field. According to the measurement data, the negative aspect is the most significant of the impact factors caused by the covering aluminum, so the sensitivity of the Hall sensor is reduced by the aluminum layer in our study.

As a paramagnetic material, when aluminum is put in a magnetic field, magnetization appears inside and the intensity of magnetic field increases slightly. Even though there is no magnetic field, atoms or molecules inside the paramagnetic materials have permanent magnetic dipoles [22]. Because of the thermal agitation, the dipoles are randomly oriented without interaction with others in the absence of an external field in pure paramagnetic materials, so there is no magnetic field inside the material. When the material is placed in a magnetic field, the dipoles will align with the applied field and a magnetic moment appears in the direction of the applied field. Generally, the influence caused by paramagnetic property is quite small and for most paramagnets the magnetic susceptibility is of the order of 10^−3^ to 10^−5^. Magnetic susceptibility is a dimensionless proportionality parameter that indicates the degree of magnetization of a material in response to an applied magnetic field [23]. Otherwise, the magnetic susceptibility for aluminum is 2.2 × 10^−5^. The current-related sensitivity increases slightly due to the effect caused by the paramagnetism of aluminum to the magnetic field. Meanwhile, the depletion region [24] caused by voltage between the aluminum layer and N-well gives rise to the reduction of the effective thickness of the active region of Hall sensor. According to Equation (2), the diminution of the thickness of the active region is beneficial to the current-related sensitivity.

**Figure 7:**
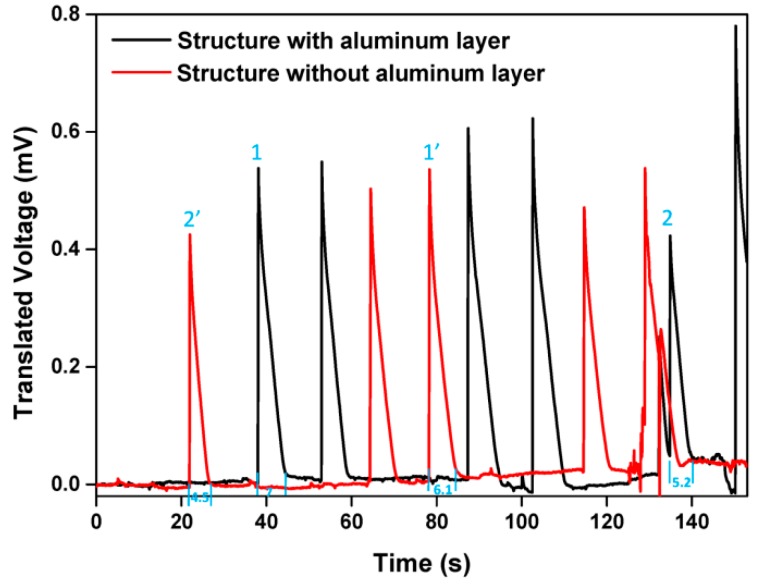
Pulses generated by the electromagnet.

Furthermore, aluminum can weaken the magnetic field because of the eddy-current effect in an unstable magnetic field. In our measurement system, the magnetic field cannot remain absolutely stable. In other words, the magnetic field fluctuates in a narrow range around the set point, so the power of the magnetic field is dissipated as a result of eddy-current loss [25]. The power dissipation of eddy currents leads to the reduction of the sensitivity of Hall sensor. In order to verify this theory, the Hall device is biased with 0.1 mA and put at the center of the electromagnet set providing a 1T magnetic field and the voltage fed back every 0.1 s. In order to analyze the difference between two structures, we translate the measured voltages around zero and two groups of the translated voltages of two corresponding structures are illustrated in Figure 7.

It is obvious that the electromagnet generates several pulses randomly. In order to ensure that the measurement data in Figure 4 is detected at the steady part of the wave, the voltmeter feeds back several results at one point and the results are analyzed to select the most reasonable one. In Figure 7, Pulses 1 and 1' have a similar amplitude, as do Pulses 2 and 2’. Pulses 1’ and 2’ coming from the wave of the structure without aluminum vanish more quickly than Pulses 1 and 2 taken from the other structure. The eddy-current effect in aluminum slows the fading of the pulses. In addition to the pulses, the magnetic field fluctuates within a very narrow range. In contrast to the pulses, the fluctuation exists in the whole measurement and is hardly discriminated in the results. This small range causes the eddy-current loss, and the power dissipation of eddy currents can be expressed by [25]:

(5) $$P=\frac{\pi^{2}B_{P}d^{2}f^{2}}{6\rho D}$$

Here *B_p_* is the magnetic field we set, *d* is the thickness of the aluminum layer, *f* is the frequency of the fluctuation, *ρ* is the resistivity of the aluminum and *D* is the density of aluminum. The values of the parameters used in Equation (5) are listed in Table 1. From Equation (5), we can conclude that the power dissipation of the eddy currents is not affected by the range of the fluctuation. Although the fluctuation is very small, it can persistently generate power dissipation that we cannot ignore in the results.

**Table 1:** Values of the parameters used in Equation (5).

*B_p_* (T)	*D* (m)	*P* (Ω·m)	*D* (kg/m^3^)
0~1	2 × 10^−6^	2.83 × 10^−8^	2.7 × 10^3^

Consequently, the magnetic field applied on the active area suffers a loss when passing the covering aluminum layer. The loss of the magnetic field leads to the decrease in current-related sensitivity. Comparing the data of S1 and S3 or S2 and S4, it is found the measurement data corresponds to the above analysis, so the Hall sensor with the aluminum covering layer suffers eddy-current loss in an unstable magnetic field and has a smaller current-related sensitivity.

From the results of a recent paper [20], the P-type covering layer benefits the current-related sensitivity, because of the decreased thickness of the active region, so in the aspect of the current-related sensitivity, the aluminum covering is not a recommendable method to reduce the flicker noise and to improve the stability of a Hall sensor.

### 3.3. Offset Voltage

**Figure 8:**
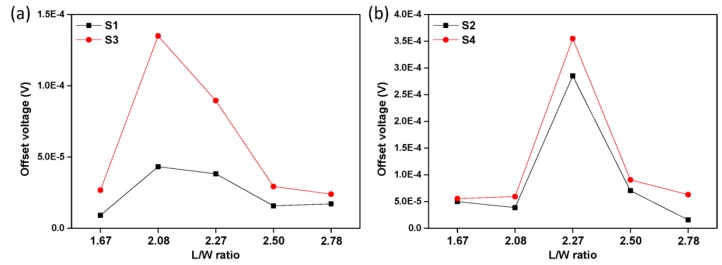
(**a**) Measured offset voltage *versus* the L/W ratio of S1, S3; (**b**) Measured offset voltage *versus* the L/W ratio of S2 and S4.

Hall sensors always suffer from a large, unpredictable and drifting offset, which limits their use in sensing low magnetic fields. To obtain the offset of a Hall device, the output voltage is the offset voltage when no magnetic field is present. Figure 8 shows the offset voltages *versus* L/W ratio of different structures and the offset voltages are measured when the Hall cells are biased by the current of 0.1 mA. It is found that the relationship between the offset voltage and the L/W ratio is not very clear. If covered with an aluminum layer, Hall sensors with the same N-type region have a poorer performance in the aspect of the offset.

**Figure 9:**
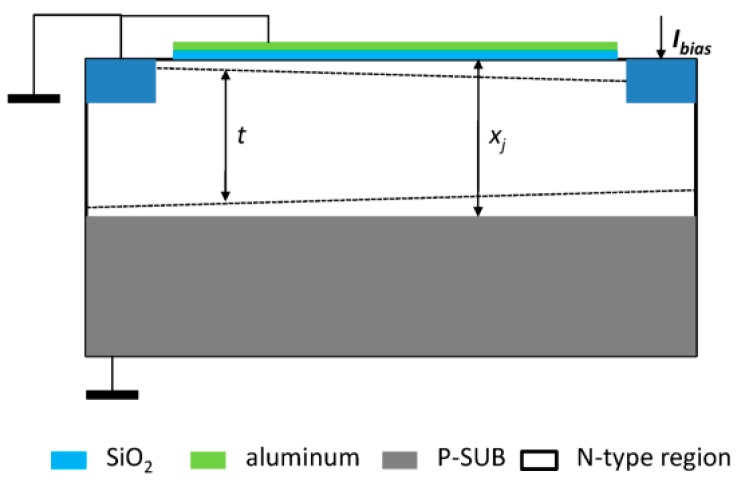
The depletion regions in integrated Hall sensors covered by aluminum.*t* and *X_j_* are the effective thickness and the total depth of the N-type region, respectively.

As shown in Figure 9, there is a reverse biased pn-junction between the P-SUB and the N-type regions. In other words, a depletion region is formed because of the voltage difference between the substrate and the active region. This phenomenon redefines the bottom edge of Hall device and is known as the JFET effect. The JFET effect always exits no matter whether the active region of the Hall sensor is covered with aluminum or not.

If the Hall sensor is covered with an aluminum layer and this aluminum layer is connected to ground, a depletion region appears between SiO_2_ and the active region of the Hall sensor due to the voltage difference between the aluminum layer and the N-type region [25]. This depletion region isolates the N-type region from SiO_2_ and forms a new top on the Hall device’s active region. The two depletion layers above and under the active region could slightly decrease the effective depth of the active region and are conducive to the current-related sensitivity. However, the offset voltage of the Hall sensors depends on the depth of the depletion region [26]. A greater depth of the depletion region leads to a larger offset voltage. It is obvious that the aluminum layer leads to a additional depletion region, which will increase the offset voltage, so S1 and S2 without covering aluminum are much better than S3 and S4 in the offset voltage aspect. In the method mentioned in [20], a P-type layer is formed above the N-type region. Therefore, one more process with the mask misalignment is necessary in the active region. The mask misalignment that is inevitably introduced by the additional process increases the offset. Moreover, the offset caused by mask misalignment is more serious than that caused by the increase of the depletion region’s thickness, so in the aspect of the offset, covering with an aluminum layer is a more suitable method to reduce the flicker noise and to improve the stability of the Hall sensor.

## 4. Conclusions

In order to analyze the influences of the covering aluminum on the current-related sensitivity and the offset voltage of Hall sensors, four different Hall sensor structures are designed and fabricated in 0.18 μm BCD lite^TM^ technology provided by Globalfoundries. Each structure is implemented in a Hall chip with multiple Hall cells. We found the impact on the current-related sensitivity caused by the aluminum covering by comparing S1, S3, S2 and S4. In an unstable magnetic field, the Hall sensor with the aluminum covering suffers a loss, which leads to a decrease of the current-related sensitivity. Moreover, the influence of the aluminum layer on the offset voltage of the cross-like Hall sensor has been studied. Due to the aluminum covering on the active region of the Hall sensor, a depletion region appears between the N-type region and silicon oxide layer. This depletion layer leads to an increase of the offset voltage. When designing a Hall device, a covering aluminum is not a perfect method to reduce the flicker noise and to improve the stability of the Hall sensor. If the current-related sensitivity is the main concern in the design, the method of covering with an aluminum layer the active layer of the Hall sensor is not a good way, however, if the offset is the most important parameter of the assessment criteria for the Hall devices, covering with an aluminum layer is a suitable method to reduce the flicker noise and to improve the stability of the sensor.
